# Sensing Sociality: Disruptions of Social Life When Living With Chemosensory Dysfunctions After COVID-19

**DOI:** 10.1177/10497323241278551

**Published:** 2024-10-10

**Authors:** Nicklas Neuman, Elin Lövestam, Jacob Karlén, Pernilla Sandvik

**Affiliations:** 1 Department of Food Studies, Nutrition and Dietetics, 8097 Uppsala University, Uppsala, Sweden

**Keywords:** biographical disruption, chemosensory dysfunctions, commensality, COVID-19, eating, food, olfactory training, sensory perception, smell, taste

## Abstract

Taste and smell are of direct importance in most social interactions. Radical disruptions in these senses can, therefore, substantially disrupt sociality. This paper focuses on the experiences of a particular type of disruption: persistent chemosensory dysfunctions after COVID-19. We conducted semi-structured interviews with 30 patients undergoing treatment for chemosensory dysfunctions and analyzed the ways in which their experiences have influenced social relations and activities, particularly regarding food and eating. The findings reveal that these dysfunctions have made the participants markedly aware that food and eating are pivotal to full participation in social life. As is smell, both surrounding smells and the perception of one’s own smell, with dysfunctions leading to several social consequences. Such problems are handled through both avoidance behavior and adaptations. While adaptations facilitate interactions, they come at the cost of feeling a burden to others or not fully appreciating an event (e.g., a shared meal). Social support is of great importance, ranging from minor practical assistance, such as a friend checking if the milk is sour, to the profound emotional relief felt from empathic treatment and recognition that the problems are real. Here, healthcare professionals can play a vital role, even in the (perceived) absence of clinical effectiveness of the treatment. The experiences expressed are partially in line with other manifestations of Long COVID and with chemosensory dysfunctions due to other illnesses, but only partially, since this is a patient group with needs and experiences that are unique, in that sociality is so strongly affected solely by disruptions in sensory abilities.

## Introduction

Sensory perceptions, such as hearing, seeing, and feeling, are of direct importance in most social interactions, as are taste and smell. Eating—for which taste and smell are cornerstones of sensory perception—is central to the organization of social life, from everyday occasions to festive events, and seems to have been so for as long as human history has been thoroughly documented (Hayden, 2014; Jones, 2007; Neuman & Jönsson, 2023). Beyond being pivotal to how we perceive food, smell also guides us in daily life—sensing if something is burning, for example, but also in subtler ways through the characteristic smells of our everyday surroundings: the subway, the forest, and the ocean. A radical disruption in these senses, therefore, implies a radical disruption in our sociality, defined as the innate human disposition toward groups. This paper focuses on the experiences of a very particular form of disruption of taste and/or smell: persistent chemosensory dysfunctions after coronavirus disease 2019 (COVID-19). We ask: In which ways have people’s experiences of such chemosensory dysfunctions influenced social relations and activities, particularly regarding food and eating?

## Chemosensory Dysfunctions and COVID-19: Prevalence, Prognosis, and Experiences

After a few months of the COVID-19 pandemic, it became clear that a distinctive symptom was loss of smell (Gerkin et al., 2021). As time passed, the profile of chemosensory dysfunctions was shown to be even broader, including not only quantitative smell reductions (anosmia and hyposmia) but also qualitative smell distortions (parosmia and phantosmia), as well as quantitative (ageusia and hypogeusia) and qualitative (dysgeusia and phantogeusia) alterations in taste (Parma et al., 2020; Vargas-Gandica et al., 2020). Currently, there is no doubt that these are key symptoms that are both more severe and persistent than similar symptoms from other respiratory infections tend to be (e.g., flu or the “common cold”) (Stankevice et al., 2023). Underlying physiological mechanisms are still debated, but several hypotheses have been proposed, some stronger than others (Butowt et al., 2023).

Prevalence estimates have varied considerably between different studies, depending on methods, populations, and time points (e.g., different “waves” of the pandemic) used. At the upper end, up to 70%–80% report some form of chemosensory symptom (of whatever form or duration) (Hannum & Reed, 2021; Santos et al., 2021). In general, objective measurements render higher prevalence numbers than subjective reports (Hannum et al., 2020). This displays the particularities of living with these symptoms; people do not know what our normal senses of taste and smell are like (compared to having or not having a fever, for example). This is noteworthy since, for some, the symptoms become long lasting.

A meta-analysis of the international literature on persistent chemosensory dysfunctions after COVID-19 estimated that around 5% still experienced some form of dysfunction at 180 days, which was the longest duration evaluated (Tan et al., 2022). The prognosis is good for the vast majority, meaning that most people no longer report symptoms in long-term follow-ups. However, the key word here is *most*; in other words, not all. In four studies based on 24-month follow-ups, all having small sample sizes and using slightly differing methodologies, a small proportion of people (approx. 3%–4%) who had not yet recovered were identified (Boscolo-Rizzo et al., 2023; Cardoso Soares et al., 2023; Deng et al., 2023; Lechien et al., 2023). Whether such chemosensory dysfunctions can be lifelong for some is still an open question, but the possibility cannot be ruled out.

The standard treatment, which is also the recommended one (Jafari & Holbrook, 2022; Levy, 2020) with some scientific support (Fjældstad et al., 2023; Helman et al., 2022; Hwang et al., 2023), is systematic olfactory training, possibly in combination with corticosteroids. However, even this treatment has a very uncertain prognosis. We are therefore far from knowing how to cure those who have not yet recovered spontaneously, which calls for research into everyday life experiences. In the present paper, we primarily target food and eating.

Some research has been carried out on this topic already, mapped out in a scoping review of food-related experiences and behavioral responses (Neuman et al., 2023). The review found, among other things, impacts on specific foods that could or could not be eaten, weight gain and loss, strategies to avoid repulsive smells and tastes, and modifications of foods to make them more palatable or sensorially stimulating (e.g., spicier or with different textures). However, there were also substantial effects on the overall quality of life and possibilities to participate socially (e.g., sharing meals and cooking for one’s family). Furthermore, the review identified a set of research gaps, namely, a lack of “in-depth qualitative explorations of specific groups, such as children and the oldest old … or those who have experienced clinical treatment for their problems” (Neuman et al., 2023, p. 396). It is the latter group which we focus on here.

## COVID-19-Induced Chemosensory Dysfunctions as a Biographical Disruption

A highly influential concept within medical sociology is that of chronic illness as a biographical disruption, originally based on interviews with people who had rheumatoid arthritis (Bury, 1982). The disruption resulting from chronic illness, it was argued, transforms social relationships, plans and expectations for the future, the taken-for-grantedness of everyday life (including one’s self-concept), the mobilization of cognitive and material resources available to the person, and how we make sense of pain and suffering (Bury, 1982). In other words, social relations and everyday life activities are abruptly transformed by the lived experiences of having an illness.

This concept has evolved considerably since its origins (Williams, 2000). A particular challenge concerns the idea of disruption itself, which assumes a “normal,” more or less linear life trajectory that all of a sudden is disturbed. For example, studying Ménière’s disease, Bell et al. suggested that a chronic illness is better conceptualized as one of many biographical oscillations that we encounter through our unavoidably messy and chaotic life course (Bell et al., 2016). This and similar criticisms (e.g., Faircloth et al., 2004) are reasonable, and clearly inferred from empirical observation. However, such critical points are not so much about the problems of the concept itself but rather about its generalizability, either across different illnesses or for specific illnesses over time. Few would disagree that the COVID-19 pandemic was disruptive, especially for those who developed long-term symptoms.

A case in point is a Canadian focus group study of 47 individuals with Long COVID (Kennelly et al., 2023). Apart from debilitating physical symptoms, such as fatigue and brain fog, the participants described anxiety of the unknown, rapid episodes of rage and sadness, hopelessness and depression (including suicidal thoughts), loss of social connection and disrupted identities, harmful experiences of social stigma, and other devastating psychosocial problems that had abruptly upset their lives. Interview studies from the United States and the United Kingdom confirm these problems (Fang et al., 2024; Spence et al., 2023). The participants struggled with getting back to normal and expressed how Long COVID resulted in a severe sense of loss in their social roles (as professionals, friends, and family) and social identities (Spence et al., 2023), profound enough to be considered a direct existential hit on one’s sense of self and being (Fang et al., 2024).

Such findings largely align with qualitative studies focusing particularly on food and eating in those living with chemosensory dysfunctions, which demonstrate acute and sudden changes in the persons’ lives as a result of their symptoms (Burges Watson et al., 2021; Høier et al., 2021; Parker et al., 2021). However, it is also important to note that if a person has persistent chemosensory dysfunctions after COVID-19, it does not necessarily mean that they also suffer from other symptoms associated with Long COVID. In fact, several experiences may actually be more similar to other illnesses that affect taste, smell, and eating functions (e.g., brain injury or certain cancers), such as the inability to cook due to sensing unpleasant smells or the social consequences of not being able to fully participate in shared meals (Belqaid et al., 2018; Bernhardson et al., 2007; Cipriano-Crespo et al., 2022). To sum up, COVID-19-induced chemosensory dysfunctions are a distinct set of symptoms derived from a new disease, one that hit like a lightning strike and paralyzed the world, creating a hitherto unknown patient group that scientists and clinicians are still, while writing, trying to understand. Our aim here is to explore how experiences of such dysfunctions have influenced social relations and activities, particularly regarding food and eating, in an unexplored population of patients undergoing clinical treatment.

## Method and Data

The data are derived from semi-structured interviews (Brinkmann, 2014) with patients undergoing treatment for persistent COVID-19-induced chemosensory dysfunctions. They were recruited from a clinic in Sweden specializing in systematic olfactory training which opened in the fall of 2021 due to the demand that emerged during the pandemic. Any patient aged 18 years or older, who spoke Swedish, and who did not report any other problems related to eating (e.g., eating disorders, major food-born allergies, or problems with chewing or swallowing), whether diagnosed or not, could participate.

### Data Collection

At a meeting with the nurse in charge of the treatment, patients were informed about the study and provided with a brief information leaflet indicating the possibility to go further using a QR code. Through the QR code, the patient was transferred to REDCap, a secure web-based system for data storage (Harris et al., 2019), in which full information about the study was provided. In REDCap, the person could provide written informed consent for participation and add his or her e-mail and/or telephone number. Those who registered were contacted by either NN, EL, or PS, and an interview was booked at a time and in a format (e.g., digitally, at the participant’s home, a café, etc.) that best suited the participant.

Thirty-one individuals registered to be contacted, of which one could not be reached. Hence, 30 people were interviewed (see Table 1 for further details about the sample). Importantly, neither the nurse nor anybody else providing treatment knew which patients had signed up for and participated in interviews. The participants were also informed that the interview study was being performed independently of their treatment. The Swedish Ethical Review Authority approved the study in May 2022 (Dnr: 2022-02353-01).

**Table 1.** Descriptive Characteristics of the Sample (*n* = 30).

Sex (M/F)	5/25
Year of birth (range)	1957–2003
Tertiary education	24
Anosmia at symptom onset^a^	26
Anosmia at time of interview^a^	7
Parosmia at symptom onset^a^	6
Parosmia at time of interview^a^	27
Ageusia at symptom onset^a^	15
Ageusia at time of interview^a^	7
Dysgeusia at symptom onset^a^	6
Dysgeusia at time of interview^a^	23

^a^Based on self-reports in the background questionnaires.

The length of the interviews ranged from about half an hour to 2 hr. They were based on an interview guide developed within the research team and in collaboration with researchers connected to the clinic. The guide had a particular, although non-exclusive, focus on food and eating and was divided into three parts: contact with and experiences of the treatment, the symptom trajectory from onset to the time of the interview, experiences and strategies, and social relationships and external support; food and beverage intake and meal routines, household responsibilities, social activities, and perceived consequences of the symptoms on their health and well-being; and thoughts about the future, such as how they thought their situation would develop and what they might need from the healthcare sector in the future. Participants also completed a short questionnaire. This included demographic information (e.g., age, educational attainment, occupation, and household composition), information about the symptoms (when they started, which ones they experienced at onset, and which ones at the time of the interview), and how long they had been in contact with the clinic. The questionnaire was marked with a randomly generated three-digit code which was used as the pseudonymized ID of each participant. In the presentation of results below, the IDs have been replaced with pseudonymized names.

### Data Management and Analysis

All data were stored in a secure cloud service hosted by Uppsala University (henceforth “the cloud”), including code keys and audio files (separated from each other). The names and contact details of the participants, however, were only stored in REDCap and therefore not in the same system as the code key. The transcriptions were performed by students who used parts of the data for their respective theses (two BSc and one MSc), and one interview was transcribed by PS. We created a specific subsite of the cloud for the transcription process in which we added audio files that were temporarily accessed by the students. Student involvement in transcription was included in the granted ethical application, and all the students were instructed about the research ethical requirements of the study. At no time did the students have access to names, contact details, or the code key. All transcriptions were stored in the cloud, as were the questionnaires with the IDs and other non-sensitive material of relevance for the study.

Data analysis involved several stages. Initially, audio files were transcribed in separate chunks by the students, for their respective theses, and by PS during late fall of 2022 and throughout the spring of 2023. This allowed NN, EL, and PS to discuss the data at different stages while proceeding with the interview process. As such, interviews were performed, findings were discussed within the group, transcribed data were checked against the discussions, more interviews were performed, further deliberations occurred, further data checks were made, and so forth. This process finally led to consensus regarding “meaning saturation” through a joint understanding of the whole material within the group (Hennink et al., 2017); the decision was then made to stop recruiting (end of March 2023). With all data transcribed, NN, EL, and PS then separately read through all transcriptions as a whole. Topics in the data were subsequently discussed and a plan for more intensive analyses with different areas of focus was devised. During this stage, NN, EL, and PS also discussed their different perspectives—the sociology of food (NN), clinical dietetics (EL), and sensory sciences (PS)—and how these may have impacted the interviewing (e.g., the choice of spontaneous follow-up questions) and the different lenses through which one sees the data. Our ambition was a reflexive, transparent, and deliberative exchange of pre-understandings and experiences in order to uphold the confirmability and credibility of the whole process (Nowell et al., 2017).

NN screened all transcribed text for data related to the research question, that is, all data including information about social relations and social activities. This reduced about 570 A4 pages of transcribed text to 192. These were then saved as a .txt file and imported into OpenCode 4.03, a tool for coding text-based qualitative data (ICT Services and System Development and Division of Epidemiology and Global Health, 2015). Since acquaintance with the data was so deep-rooted within the research group already at the start of coding, several empirical patterns were fleshed out by initial analyses of transcribed chunks of data. This is also when theory (i.e., illness as a biographical disruption) entered the analysis, since we looked for conceptual tools to understand the rapid disruptions in the lives of patients. The coding is therefore best described as a top-down process—not deductive in the conventional sense of testing whether a theory is false or not (Hyde, 2000) but to check for confirming or contradicting evidence of both theoretical and pre-established working hypotheses about the data (Casula et al., 2021; Gilgun, 2014, 2019). For example, it was clear to us early on that the participants’ symptoms affected their social needs and their (perceived) social influence on others. The detailed coding helped us explore these working hypotheses and determine more exactly how such needs or perceived influences were manifested (or not). Examples of codes relating to this are “Being a burden,” “Affecting close relatives,” “No one understands,” “Not alone with the problems,” “Recognition (it’s real),” and “Comparisons with others.” As such, the process was iterative, with working hypotheses about the data continuously checked and findings modified thereafter.

## Findings

As expected, the experiences of participants varied enormously. When the severity of symptoms was milder, so were the impairments on social life generally. Commonly, things were described as being disrupted “overnight.” All of a sudden, one realizes at the hotel breakfast, at lunch in a colleague’s house, or when throwing away trash in the garbage room that one’s sense of smell or taste is gone, significantly diminished, or distorted in a repulsive way. From this moment on, life is very different, affecting social relations and activities in several ways.

### Awareness: “Everything Revolves Around Food”

There was a clear tendency among participants to describe their problems as almost a social wake-up call, or at least as an intense reminder about how incredibly central food and eating are for social relations and activities, but also how present and significant smells (including one’s own) are in society. Mona was a woman in her mid-40s who suffered from severe taste and smell distortions that gravely affected her everyday life. She was repulsed by almost all foods, toothpaste, shampoo and conditioner, and the smell of her towel. Even the smell of her husband and children was difficult to handle. She described how “you realize how much you actually [Laughs], via the food, how much of your well-being is there … mostly you celebrate with food, it’s often what you turn to for something extra festive, nice, or cozy.” “It consumes you,” she said, “it really does.” Moreover, “to feel a bit normal,” she would sometimes have a cup of tea with milk for breakfast or at the office, although “it doesn’t taste good, but it doesn’t taste awful,” which coffee did. This, she continued, was “mostly just to feel a bit like you’re used to, that you’re kind of walking around with a cup and participating.” Hence, since tea with milk was not awful, she could drink it as part of normal social interaction.

As another example, Agnes, a woman in her sixties with distorted smell and reduced taste, was asked about how she handled big holidays: “Horrible, horrible. … Christmas I think is, it’s really horrible … No, no, it’s not any fun with, with everything that just, just revolves around food all the time. Then I feel extremely left out and alone.” A noteworthy addition to this is her reasoning concerning the most recent Christmas celebration prior to the interview, which occurred in her new house. In the new house, “I’ve never had any [sense of] smell and taste, so the [former] house was also associated with a lot of before and after,” and she went as far as thinking that this emotional detachment from established Christmas traditions may have played a part “in saving me from falling into a depression.” As such, the lack of chemosensory reminiscences of a physical location made sociality more manageable.

Another topic raised in several interviews concerned romantic encounters, both dating and established romantic relationships. For example, Pia, another woman with both distortions and reductions in taste and smell, talked about how dating was compromised by having to worry about her sense of smell and being unable to enjoy a meal. For example, dinner dates including wine could be a problem, since wine caused an intense and unpleasant smell in the room (described as acetone). She was asked whether she had developed any strategies, such as choosing other activities unrelated to food.

I haven’t [Laughs]. But, it becomes unsustainable in the long run. Like at first you could, yeah, go for walks or do other activities together … But in the end you eat three times a day. So you can’t avoid food.

She went on to speak about social interactions more broadly, beyond dating:

That’s what’s been most depressing, or something I realized, that I don’t think people with normal smell get … that everything in society is about food. Like everything you do socially … revolves around food. You go to parties, you’re offered food, you go to restaurants. Like when you hang out with people you can’t not eat. Even if you can spend a few hours doing some other activity, then it’s: “Let’s go for fika!,” “Let’s have lunch!,” or “Let’s have dinner!”

*Fika* is a social institution in Swedish culture (Yngve et al., 2023). It is difficult to translate but can be described as a social practice centered around taking a break to socialize over coffee or tea, sometimes with a savory or sweet snack. As any person who has ever experienced Swedish culture probably knows, not being part of fika is to be left out of a core institution for social interaction. Social relations and social activities will sooner or later, following Pia’s line of reasoning, include fika. They will also include lunches and dinners, since “everything in society revolves around food.”

In a similar way, Julia, a woman who was approaching thirty and reported more or less complete loss of both smell and taste, reasoned about social interaction in adulthood, where “a lot more revolves around [food]” compared to when she was younger. Previously “it was more like movie nights,” whereas now “it’s like, over dinner … the focus [nowadays] is often on meeting for food or drinks.” She did not avoid social activities, since “that’s where I get my energy from,” but the symptoms had opened up a plethora of problems, such as not knowing what to say when someone at a restaurant asked if the food was good or not being able to truthfully tell her friends that she had enjoyed a meal. “I don’t want to lie either,” she said, sobbing slightly: “Things like these, I really struggle with … if a friend or someone treats me to food then I have real trouble saying ‘Thanks, it was delicious’ because I don’t know.” She was not alone with this dilemma of having to choose between lying and not expressing gratitude. Ellinor, a woman in her early sixties with both senses more or less absent but also distorted, expressed a similar sentiment. When invited out, she sometimes said she enjoyed the food despite having “no idea” about the taste, saying that “it’s really tough, like, having to explain all the time … so sometimes I just make something up, like, I can’t stand to fight that battle.”

All in all, the experiences of persistent chemosensory dysfunctions have led to a revelation about the pivotal place that food and eating have in life, meaning that the inability to experience smell and taste in the same way as before hampers social interactions. Any prolonged social relationship, whether with family, colleagues, friends, or romantic partners, will inevitably involve food: either as a guest, host, or simply as a part of the group during everyday activities. As shown in the next theme, when these were impaired, they were managed by either avoidance behavior or different forms of adaptation.

### Avoidance and Adaptation: Isolation, Burden, and Boredom

A radically disruptive way of handling the chemosensory dysfunctions in relation to social life was to simply avoid situations that were either unmanageable or felt pointless. However, the extent of the avoidance behavior differed. Let us demonstrate this by juxtaposing Penny and Felicia. Penny, a woman in her mid-20s with reduced and distorted taste and smell, was asked how she had handled meals with others, responding: “I’ve avoided those.” She continued:

[I] haven’t been able to join others at a restaurant, or things like that, when the parosmia was at its worst. … And like, at work I even avoided being in the staff room because there were so many smells. You absolutely isolated yourself.

Consequently, when the symptoms were at their most severe, she isolated herself. Sometimes, however, she could eat a few things, “then you can say ‘Yeah, but can we choose this restaurant which I know has things I can eat?’,” exemplifying an adaptation that could reduce the isolation although still being very different to social life before. Felicia was about a decade older and reported similar symptoms. In contrast to Penny, however, she did not depict a very impaired social life. When asked, she could only think of one occasion when she and her partner planned to visit “some posh Michelin restaurant.” “[I]t was quite expensive,” she said, “so I felt it was really quite unnecessary [to go] actually. So uhm, my partner went with someone else instead, because it would have felt like throwing money down the drain. But other than that, no, nothing else [has been avoided].” Felicia could have probably managed the restaurant visit had she wanted to; it was not repulsion that seemed to stop her. Rather, there was simply no point in spending money on fine dining without being able to taste the food properly.

As already touched upon in the previous theme, another form of special occasion that could cause trouble was holidays. Birgit, a woman in her sixties with distorted taste and smell, spoke about the Christmas season and “all the mingling with mulled wine, Christmas lunches, Christmas buffets, and everything.” She found it “really sad to say no to everything and everyone.” The previous year, she and her husband had chosen to go abroad for Christmas, which they were planning to do again, or as she put it, “[W]e fled the country [Laughs] to escape the Christmas hysteria, because everything revolves around food.” We also interviewed her husband Fred, a man of the same age who reported almost identical symptoms. He confirmed the avoidance behavior, saying that they refrained from visiting other peoples’ homes to avoid repulsive food odors. Asked if the strategy had altered from the onset of the symptoms and onwards, he responded:

I think it’s the same. Like, it’s still the same problem so we haven’t, we’ve just avoided visiting people. Summer has been good, when you can socialize outdoors, with the children and grandchildren. But later on, you can’t meet for fika and things, not now. But you could in the summer if you could sit outside.

Birgit and Fred had to isolate together since unpleasant tastes and smells prevented them from engaging in social interactions. However, interacting outside when the weather allowed was one way of modifying social circumstances to make them manageable. This form of less disruptive adaptation to the new situation was indeed common. Maria, a woman born in the mid-90s who reported distorted taste and smell, as well as reduced taste, serves as an example. To her, commensality of the family was imperative. This included eating, if not the same, then at least similar things. “We try to eat the same food,” she said, “If they eat pasta and meatballs then I eat pasta and mushrooms. So it looks similar at least.” She was then asked what it is like to sit and eat with others, given the smells, to which she answered:

I mean, there aren’t a lot of alternatives [Laughs]. To me, dinner time, dinner time especially, is very important. It has been since I was a child because that’s when you gather as a family at eye-level with each other. Yeah, then I can’t sit and complain [Laughs].

Importantly, the possibility to adapt was not described as being unequivocally positive. While it facilitated interactions, it came with certain costs. One of which, hinted at in Maria’s comment that she “can’t sit and complain,” was a concern that several participants voiced: of being a burden to others, due to their restricted eating. Mona talked about the inability to fully enjoy a festive event or cozy occasion with significant others and about feelings of being a burden to people who tried to conform to her situation. For example, when her husband “cooks really lovely food for [the children] and also makes something for me … you see in him that they don’t want to fully enjoy the food.” This is an adaptation where her husband cooked differently for her and the rest of the family, and hence she could participate socially on her terms. Still, the social consequences, as we can see in the quote, were a feeling of guilt for how she, as she perceived it, indirectly hampered her family’s enjoyment of the food.

Eva, a woman in her mid-40s with taste and smell distortions who avoided some social situations, also described adaptations, such as meeting people outside, culinary modifications at home while cooking for others, or bringing her own food to dinners. It could also be specifically choosing restaurants that worked for her, like sushi, “because there’s rice, salmon, and avocado,” and no onions, which were impossible. “I was an extremely annoying guest,” she said, “when invited round to others I’d rather say ‘Yeah, I’ll bring my own food, cook whatever you want [Laughs] for the rest of the group’.” Similarly, after talking about how daily food routines were affected, Lisa, another woman born in the mid-90s who had distortions and reduced smell, was asked whether she currently went to dinners as she used to: “Yes … But I’ve never avoided that. It’s just that I’ve felt [I was] a bit annoying sometimes.”

Lastly, in addition to feeling a burden, adaptations could simply make social activities dull. When talking about restaurant visits, Rebecca, a woman about 50 years old, with reductions and distortions of both senses, said at the time of the interview that her taste perception had returned somewhat. At the onset, she could not sense anything, but “now I can find [restaurant visits] quite nice.” Before this, however, she had both avoided them and adapted by participating without having to eat anything. Instead, she joined the group over a glass of wine.

I could even be like, “I’ll join and have a glass of wine, but I won’t eat,” because there’s no poi-, it’s not fun. So then I was like, “I can come along as company,” uhm, just have a glass of wine and chitchat a bit. Really weird maybe. But then, at least you joined in but maybe didn’t eat the food, but that’s really boring too of course.

What the quote above suggests, as do several of the stories touched upon so far, food and eating are the vehicles for social activities and therefore meaningful relationships. Sensory impairments—both reduced and distorted—have consequences for the maintenance of such relationships. However, as we will see below, social relations are also pivotal resources of support, both practically and emotionally.

### Assistance and Support: Practical Help and Emotional Relief

We now turn to questions about the role of social relations as resources for the participants, including the potential difficulties when they were lacking. To begin with, one such resource regarded access to simple, practical help in everyday activities that are normally guided by taste and smell. Isabelle, for example, was a woman in her fifties with both senses reduced and distorted. She said, “if I’m about to cook or smell if something, if milk is sour for example, I can’t detect that so I have to ask for help to judge those things.” She further described this need for assistance as “a bit debilitating, or how do you say, that you can’t identify if food is bad.” This is common in the data. People in the participants’ immediate social surroundings had to assist in checking how food tasted or smelled, or whether their bodies or clothes smelled like sweat, and so forth. This was also described by Jasmine, a woman in her early twenties with distorted smell, and both reduced and distorted taste. When asked about how living with the chemosensory dysfunctions had affected her health (physically, mentally, and emotionally), she said:

… it has affected [me] mentally, like you develop a mindset that you didn’t have previously around food, and like I’ve had a bit of an issue with washing clothes and those kinds of things, and it’s been like “Now I’ve used this sweater once, can I use it again?”, sort of. I’ve been like, sometimes I have actually asked my mum or somebody “Does this smell weird?”. Like, you have to use other people. … [I]n that way I’ve become insecure and like … perfumes and stuff, I’ve been like “You have to smell this,” like, “can I wear it?” A lot of that stuff … in relation to others, it can be burdensome sometimes.

As these two quotes demonstrate, for some this was expressed as inconvenient, “a bit debilitating” as Isabelle described it, or more socially burdensome as for Jasmine. However, it is also easy to see how such a need for assistance can have more damaging consequences if it is absent. The inability to identify spoiled food is a potential health hazard, and with nobody around to identify smells for you, nobody can sense if something is burning on the stove or if there is a fire, for example, as noted by others.

For Lisa, one practical way of handling this was to try things out around people she trusted, like her mother or friends, providing a safe environment to experiment in without any financial cost, pressure, or sense of guilt. If it turned out that the taste of a food had once again become manageable “then I can like, start buying it again.” Furthermore, Alexandra, a woman in her early thirties with significant reductions and distortions of both senses, also told a story about cozying up with her small nephew when her sister told her that he had defecated. She had not noticed, and this inability to smell a dirty diaper was expressed as a problem by others as well. Alexandra laughed about the situation, so there were seemingly no serious ramifications in her case. While some situations can indeed be humorous, not having someone close telling you about the need for a diaper change can be both a sanitary hazard to the child and a very severe social stigma for the responsible adult.

Social resources also meant emotional relief. In the quote below, Lisa describes a trajectory from finding out through social media that she was not alone, which had some instantly supportive effects, whereas long-term resources came from significant others, in particular her boyfriend who had taken it upon himself to cook her food she could eat.

In the beginning, when I finally understood what it was and searched for Facebook groups and realized that there were Facebook groups, and some of them have had COVID. That in itself was sort of a support. Not that I’ve received a lot of support from them personally, but it was supportive to know that it exists and a really big support to know that everyone had the same [problems]. … But then I’ve had huge support from like, yeah family and … my boyfriend there at the start. It was amazing that he, he like took it as a challenge and thought it was fun [to cook for her]. Then of course, after a while he got tired of it, “Can’t there be a tiny bit of onion?” But it has been really, really good. He’s, he’s been amazing with this.

The description of support from social media is fairly representative. It could be encouraging, at least at the start, to feel a recognition of not being alone, but then the activity diminished, and several participants had no real experience of receiving support from social media. Clearly, for both Lisa and other participants, it was significant others who were the most important source of emotional support. Birgit even said that if Fred, her husband, had not shared the same problems “we probably wouldn’t be living together today, I tell you.” Nobody else could understand, she figured, and “if he had eaten normally I wouldn’t have been able to stay in the same apartment.”

While this form of emotional relief is less acutely important compared to the ability to detect spoiled food or smell a fire, the lack of it can still be damaging. John, a man in his mid-30s whose smell and taste were still mostly absent at the time of the interview, almost a year after onset, spoke about experiences of lacking support. At the start, people in his surrounding environment did not find his symptoms strange or anything to worry about, he said, but this would change:

With time I think, it’s been like, you’ve felt very like, unappreciated by your surroundings. They sense a smell that everyone else is sensing too, and then I’m there and can’t smell it. It’s like hard for them to take me seriously when I say “But I really don’t smell that,” and it’s like you become, yeah, I wouldn’t say they were disparaging towards me but I was a bit like declared dumb by some. … So uhm, but no one was like disparaging towards me, most people have been quite nice but … many have a complete lack of understanding about the whole, like [situation].

This feeling of being questioned or not understood is expressed very tellingly by John’s words, but also expressed in different ways by several others. Alexandra explained further how she could get positive comments from people who celebrated that she had sensed a taste or a smell. While well-intentioned, this made her angry, and she said: “Because I have told them a hundred million times that … it’s still gross. Nothing tastes good. … And I have explained it to everyone, ‘You have to understand that I’ve graded my food as either very gross or slightly gross’.” Thus, questions and comments, even positive, could be unsupportive in that they so clearly manifested a lack of understanding about the lived reality of the symptoms.

In instances when support from significant others was absent or insufficient, an important source of emotional relief could also come from clinical professionals. Shortly after her account which was quoted earlier, Lisa talked about supportive healthcare encounters and how important it was for her to be followed up and taken seriously, “even when it hasn’t, like, changed much [with the symptoms].” Agnes described this more clearly when asked about how she had experienced the consultation with the nurse at the clinic:

It felt really good to … talk about this, with a person who, it felt good to have it played down a bit. This was her job, she talks to people about this every day I guess … it was nice too that it wasn’t like “Oooh!” you know like it can be at work, people come and ask [questions] sometimes. I’ve spoken to some people about it but now, now I feel like, I can’t be bothered anymore … But this nurse, she … it wasn’t dramatic …

Stories like those from Lisa and Agnes testify to the pivotal role that healthcare professionals can play in social support. Importantly, in the data, there is also a lot of disappointment with the healthcare system, and the experiences of meetings with nurses, dietitians, and doctors vary considerably, but when they worked well, it was expressed as a source of support. Agnes spoke further about how the nurse had validated her and given her positive feedback on her olfactory training, but on a direct question about whether the symptoms had improved, the answer was plainly: “No.” Yet despite this, the nurse was praised. Her empathy was the resource, in showing that she recognized what Agnes was going through, that she believed her, and that she helped by playing down the situation while offering care. However, to be clear, some participants reported that they thought the olfactory training had helped. The point here is not to evaluate the clinical effectiveness, but only to show that regardless of clinical outcome, the experience of being understood, taken seriously, and listened to could provide emotional relief.

## Discussion

This study has provided insights into everyday social relations and activities of individuals undergoing treatment for persistent chemosensory dysfunctions after COVID-19, based on semi-structured interviews with patients from a specialized clinic in Sweden. The dysfunctions have made the participants markedly aware that food and eating are pivotal to full participation in social life. As is smell, both smells in their surroundings and the perception of their own smell, leading to social consequences related both to the inability to identify smells (when reduced) and the inability to manage repulsive smells (when distorted). Such problems are handled through both avoidance behavior—when it becomes too difficult or feels pointless—and adaptations (e.g., meeting people outdoors, picking specific restaurants serving foods that work, or eating or drinking different things to one’s meal companions). While adaptations facilitate face-to-face social interactions, they come with the costs of feeling a burden to others or not fully appreciating the event. Of great importance is social support, from minor practical assistance, such as a friend telling you if the milk is sour, to profound emotional relief from empathic treatment and recognition that their problems are real. Here, healthcare professionals can play a vital role, even in the (perceived) absence of clinical effectiveness of the olfactory training. In the following section, we reflect on the main contributions of the paper and the study’s strengths and limitations.

Social analyses of taste have a long history, primarily following the tradition of Bourdieu (1979/1984). However, taste in that sense is about cultural distinction and symbolic expression, which means that food and eating are treated as markers of social status in the same way as classifications of “good” or “bad” taste relate to other forms of esthetic consumption (Gronow, 1993; Warde, 1997). Our analysis deviates profoundly from this tradition, in that we have analyzed the actual *sense* of taste and its profound social significance in and of itself (cf. Højlund, 2015). Given the multisensory nature of food perception (Auvray & Spence, 2008), this social significance is equally intense for smell. Consequently, our findings demonstrate how chemosensory functions are pivotal to the structuring of social interactions and social relations. We consider this a relevant insight for anthropologists and sociologists interested in food, eating, and taste and suggest that regardless of theoretical tradition, sensory perception has a place in social analysis.

The centrality of food and eating in social life in general (Gronow & Holm, 2019; Neuman, 2019; Warde, 2016) and the social significance of the shared meal in particular (e.g., Fischler, 2011; Jönsson et al., 2021; Kerner et al., 2015; Simmel, 1910/1997; Sobal, 2000) are widely documented. That this would, in some way, be confirmed by our data was therefore anticipated. We were, nevertheless, surprised to find how this was almost a revelation for some: how “everything” in social life and society revolves around food, which could lead to feeling isolated. This appears to be an effect of experiencing the sociality of food in a way that is out of the ordinary, as realized by people who abruptly lost their ability to participate in normal food-related activities, such as the Swedish fika. Disruptions in life, whether due to illness or something else, are therefore particularly suitable for analyses of the commonsensical habits, routines, and rituals of everyday life, whether food-related or not. Our vision of our social surroundings is never as clear as when we are suddenly hampered in our ability to participate and find ourselves observing them from the outside.

Closely connected to this possibility to participate socially are the resources available from one’s social network. From a generalized point of view, the need for social support, and the detrimental consequences of a lack of this, is not a surprising finding for health researchers (Uchino, 2006; Wang et al., 2003). Even so, our study adds details about how such support can be given and what it can entail for this particular patient group, both as practical assistance and emotional relief. For several participants, one form of relief could come from social media, in the recognition of not being alone and of the experiences being real. This source of support has been observed elsewhere, both for Long COVID in general (Rushforth et al., 2021; Russell et al., 2022; Thompson et al., 2022) and its chemosensory symptoms in particular (Burges Watson et al., 2021; Parker et al., 2021). It should, therefore, not be trivialized, yet social media seemed inadequate in and of itself and secondary to significant others. We can also see that clinicians who, apart from providing the standard treatment, listened to, believed in, and approached the patients with respect and empathy, contributing another valuable form of emotional support. Lastly, since previous research has identified associations between chemosensory dysfunctions and major depressive disorders (Liu et al., 2022), mood disorders (Llana et al., 2023), and lower quality of life (Winter et al., 2023), further professional psychological support may be required (Neuman et al., 2023).

As mentioned in the background of this paper, we argue that we have studied a condition that is exemplary as a biographical disruption: an identifiable set of symptoms appears and abruptly influences social relationships and patterns of social activities, to very different degrees, but abruptly nonetheless. An obvious contrast to many other health conditions that are described as causing biographical disruptions (or neighboring terms) is the lack of shared understanding of the situation. Rheumatoid arthritis (Bury, 1982), stroke (Faircloth et al., 2004), and different forms of disability (Larsson & Grassman, 2012) have been known for much longer. They are, therefore, attached to strongly held ideas of who suffers from them, whether or not one “deserves” them, and much else. Clearly, during the pandemic, much has been learned about taste and smell too, and the participants spoke about different levels of awareness among the people around them. However, this is still different to more commonly known illnesses and health conditions, as is evident in the expressed experience in our data about uninformed questions and comments. The way we interpret an illness or a disability depends on what we have learned previously, so it is not surprising that some would expect taste and smell to become normalized between one interaction and another. This is what people are used to, from having lost or having experienced distortions during a cold, the flu, or even COVID-19. This is probably an important aspect of why social recognition of their experiences was so important to some.

We must also keep in mind the experiences of this patient group compared to the core symptoms associated with Long COVID, such as fatigue, brain fog, and cardiovascular manifestations (Fang et al., 2024; Kennelly et al., 2023; Spence et al., 2023), and the chemosensory dysfunctions caused by other illnesses (Belqaid et al., 2018; Bernhardson et al., 2007; Cipriano-Crespo et al., 2022). Our participants reported experiences that were similar to and different from both. If chemosensory dysfunctions were the sole symptoms of Long COVID, the general repertoire of health issues would be very different, but this does not mean that they can be trivialized to “just a bit of smell loss.” As we have seen, and as previous research has shown, the psychosocial consequences can be detrimental (Liu et al., 2022; Llana et al., 2023; Neuman et al., 2023; Winter et al., 2023), and restrictive eating can have nutritional consequences. Moreover, being repulsed by food odors can be a shared problem for many with distortions, regardless of cause, but the broader consequences for social life and activities will inevitably differ depending on whether this is one’s only disability or one of several manifestations of a deadly disease. The clinical and public-health implications are such that we cannot straightforwardly transfer experiences from other patient groups (e.g., Long COVID in general or cancer patients with chemosensory dysfunctions) to those of this study. Consequently, in treating and supporting this patient group, we cannot assume that their requirements are the same either.

### Strengths and Limitations

A strength of this study is the unique sample, providing insights into the everyday life of people whose symptoms have been confirmed as severe enough to justify clinical treatment rather than based on self-identified symptoms. The approach also offers new knowledge about their experiences of the relationship with the healthcare system, including specialized olfactory training. A possible drawback concerns selection problems. Access to the clinic depends on which Swedish region the individual is registered in. In addition, for people within eligible regions, we must also acknowledge potential inequities in access and awareness of one’s rights. Among the patients being informed about the study at the clinic, there may also have been selection bias regarding participation. While the skewness in terms of gender and age largely follows what would be expected from the overall prevalence numbers, the educational level of the sample suggests a bias. The fact that the study is explicitly about food and eating, and that several of the questions targeted this specifically, must also be acknowledged. If the focus had been broader, with no explicit food focus, the results might have been different. There is no reason to believe that the accounts were untrue, but other aspects of the participants’ lives may have been given more space had the study been designed differently. The composition of the research group is a strength, including competence in the sociology of food, dietetics, and sensory science, as well as experience in qualitative methods. So is the structured, collective process of analysis, including three student theses providing spot checks of the data that could later be used for formulating working hypotheses to be explored deductively in the main analysis.

## Conclusion

In conclusion, the ways in which experiences of COVID-19-induced chemosensory dysfunctions can influence social relations and activities, especially regarding food and eating, concern everyday awareness and management of sociality, and the resources one draws upon to handle day-to-day struggles and abnormalities. New conditions for sociality appeared abruptly, and the experiences are partially in line with other manifestations of Long COVID and with chemosensory dysfunctions in other illnesses. Though, only partially, since this is a patient group with needs and experiences that are unique, in that sociality is so strongly affected solely by the disruptions in their sensory abilities.
